# Efficacy of Cefpodoxime with Clavulanic Acid in the Treatment of Recurrent Pyoderma in Dogs

**DOI:** 10.1155/2014/467010

**Published:** 2014-01-12

**Authors:** B. Sudhakara Reddy, K. Nalini Kumari, V. Vaikunta Rao, V. C. Rayulu

**Affiliations:** ^1^Department of Veterinary Medicine, College of Veterinary Science, Sri Venkateswara Veterinary University, Tirupati, Andhra Pradesh 5170502, India; ^2^TVCC, C.V.Sc., Proddatur, Andhra Prandesh 516360, India; ^3^Department of Veterinary Medicine, C.V.Sc., Tirupati, Andhra Prandesh, India; ^4^Department of Veterinary Medicine, C.V.Sc., Gannavaram, Andhra Prandesh, India; ^5^Department of Veterinary Parasitology, C.V.Sc., Proddatur, Andhra Prandesh, India

## Abstract

In the present study on recurrent pyoderma, dogs with a history of more than three episodes of skin infections in a period of one year were selected. The associated conditions and (or) underlying factors revealed upon thorough investigation were demodicosis, *Malassezia* dermatitis, flea infestation, hypothyroidism, keratinization disorder (seborrhea), combination of *Malassezia* dermatitis and tick infestation, and a combination of scabies and tick infestation. Therapy was given with cefpodoxime with clavulanic acid along with appropriate simultaneous medication for the underlying associated conditions. In all the cases response to therapy was excellent. Improvement was noticed by 9 to 19 days and 17 to 21 days in recurrent superficial and deep pyoderma, respectively. In one dog, relapse occurred by 45 days due to the associated condition of hypothyroidism which was confirmed through laboratory findings. Cefpodoxime with clavulanic acid proved to be an effective, safe, and convenient antibiotic for the treatment of recurrent pyoderma in dogs without any side effects.

## 1. Introduction

Dogs suffer from a variety of skin infections. Canine pyoderma is one of the most common diseases. Pyoderma literally means pus in the skin and can be caused by infectious, inflammatory, and/or neoplastic etiologies; any condition that results in the accumulation of neutrophilic exudates can be termed pyoderma. Most commonly, however, pyoderma refers to bacterial infections of the skin. Pyodermas are common in dogs and less common in cats [1]. Pyoderma classified according to the depth of infection into surface, superficial, and deep pyoderma. Surface pyodermas are those infections that are restricted to the surface of the skin and not extended into the follicle; it does not extend deeper than the stratum corneum or into hair follicle. Superficial pyodermas include infections that involve the hair follicle but do not extend into the dermis. Deep pyodermas are infections that extend into the dermis and underlying panniculitis [2]. Pyoderma is caused most frequently by staphylococci. Bacterial pyoderma is usually triggered by an overgrowth/overcolonization of normal resident or transient flora. Among different pyoderma conditions, recurrent pyoderma is an important clinical skin problem and frequently occurs as a result of uncorrected underlying cause(s) or use of inappropriate antibiotics or improper duration of antibiotic therapy. Recurrent pyoderma is the infection which responds completely to an appropriate therapy leaving the dog apparently normal between episodes of infection. Most common persistent underlying skin diseases in pyoderma include nonparasitic allergic diseases (atopic dermatitis and food allergy), parasitic allergic diseases (flea allergy, scabies, and cheyletiellosis), demodicosis, endocrine diseases (hypothyroidism and hyperglucocorticoidism), diseases of cornification (primary cornification defects and secondary seborrhea), other infectious skin diseases (*Malassezia* dermatitis and dermatophytosis), genodermatoses (follicular dysplasia, colour dilution alopecia, and sebaceous adenitis), occult neoplasia (solar-induced squamous cell carcinoma), and immunodeficiency (congenital and acquired) [3]. Cephalosporins are often used to treat canine skin infections because of their broad antimicrobial spectrum, established safety profile, and reasonable cost. Cephalexin, cefadroxil, and cephalothin have all been recommended for use in treating canine pyoderma. Cephalexin and Cefadroxil need to be administered orally twice daily for therapeutic purposes. Recently, cefpodoxime proxetil was approved for treating skin infections in dogs. Once daily administration sets it apart from other oral cephalosporins used in veterinary medicine. Hence in the present communication, a detailed outcome of use of cefpodoxime with clavulanic acid in the treatment of recurrent pyoderma in ten dogs of one-year research period is reported.

## 2. Materials and Methods

The present investigation was carried out on the dogs referred to C.V.Sc, S.V.V.U., Tirupati, Andhra Pradesh, India, and dogs presented at the major veterinary hospitals around Tirupati. Dogs with a history of more than three episodes of skin infections in the past one year were included in the study as also done by Bensignor and Germain [4]. The dogs were thoroughly examined clinically for the presence of macroscopic ectoparasites. Laboratory examination was carried out by glass slide impression smears, tape impression smears, skin scrapings, and hair plucks as per the methods described by Curtis [5] and Rosenkrantz [6] in order to confirm pyoderma and other concurrent dermatoses. Whole blood and serum were also collected for studying haematology and serum biochemistry in order to find out or confirm the associated conditions and (or) underlying factors. During the one-year period of dermatological examination on dogs, 13 dogs were found having recurrent pyoderma. Details about age, sex, breed of different dogs, type of pyoderma, duration of infection, and previous antibiotic therapy were mentioned in Table 1. All the dogs were treated with cefpodoxime with clavulanic acid at 5 mg/kg body weight, once daily, orally [7] and the antibiotic was continued up to one and two weeks beyond the point of clinical recovery in dogs with recurrent superficial pyoderma and recurrent deep pyoderma, respectively. The dogs were monitored clinically at regular intervals, that is, on days 7, 14, 21, and 28, and so forth after therapy. Efficacy of therapy was assessed based on the reversal of symptoms and attainment of clinical normalcy. Response to therapy was graded as excellent, good, fair, and poor by assessing the clinical symptoms and lesions [8]. In all the dogs, time taken for complete recovery was also noted as per owners' statement. All the dogs were monitored for a period of six months after recovery for recurrence of pyoderma. Supportive therapy was carried out with daily supplementation of a skin tonic, that is, glossy coat, at the dose rates suggested by manufacturers and bathing was advised twice weekly with benzoyl peroxide (2.5%) shampoo. Recurrent pyoderma associated with demodicosis was treated with oral ivermectin at 300–600 *μ*g/kg body weight as incremental doses, looking for any toxic symptoms [9]. Treatment was continued till two consequent negative skin scrapings were obtained at an interval of ten days. Scabies was treated with oral ivermectin at 200 *μ*g/kg body weight, twice weekly till the skin scrapings became negative besides clinical improvement. Ticks and lice were treated with ivermectin at 200 *μ*g/kg body weight, subcutaneously once, followed by external application of cypermethrin once a week to prevent recurrence of external parasitic infestation. Flea infestation was treated with fipronil spray twice a month [10]. Hypothyroidism was treated with oral Levothyroxine sodium at 20 *μ*g/kg body weight twice a day. *Malassezia* dermatitis was treated with oral ketoconazole at 5 mg/kg body weight per day given along with food and treatment for seborrhea was carried out with skin tonic containing essential fatty acids.

## 3. Results and Discussion

In the present study, out of 13 dogs selected, seven were females and six were males. Recurrent superficial pyoderma and recurrent deep pyoderma were noticed in ten and three dogs, respectively. Age of dogs ranged from 1 to 8 years, with breeds like German shepherd, Labrador, Lhasa Apso, Dachshund, Rottweiler, Bullmastiff, Doberman, Pug, and nondescript and weights ranging from 8 to 40 kg. Duration of clinical signs ranged from 3 to 18 months. The dogs were previously treated with different antibiotics such as penicillin, lincomycin, enrofloxacin, amoxicillin clavulanate, cephalexin, and amikacin but for a shorter period of about one week. Thorough anamnesis revealed that failure to identify and treat the underlying factors, use of a narrow spectrum or an inappropriate antibiotic(s), and therapy of an insufficient duration might be responsible for recurrent pyoderma in the dogs presented. Upon thorough investigation, it was found that, out of 13 dogs with recurrent pyoderma, demodicosis, *Malassezia* dermatitis, flea allergic dermatitis, and hypothyroidism were noticed in 2 dogs each followed by keratinization disorders (seborrhea) in one dog. Mixed conditions, that is, combination of *Malassezia* dermatitis and tick infestation, and a combination of scabies and tick infestation in one dog, were also noticed. However, no associated conditions could be noticed in the remaining 2 dogs. Simultaneously, Bloom and Rosser [8] failed to identify the underlying cause associated with pyoderma in 2 dogs out of 21 dogs. Bensignor and Germain [4] also could not identify the associated conditions in two out of 30 dogs of their study on canine recurrent pyoderma. Though other biochemical tests for detection of any underlying Cushing's disease were not carried out, no dog was clinically suspected for it.

**Figure 1:**
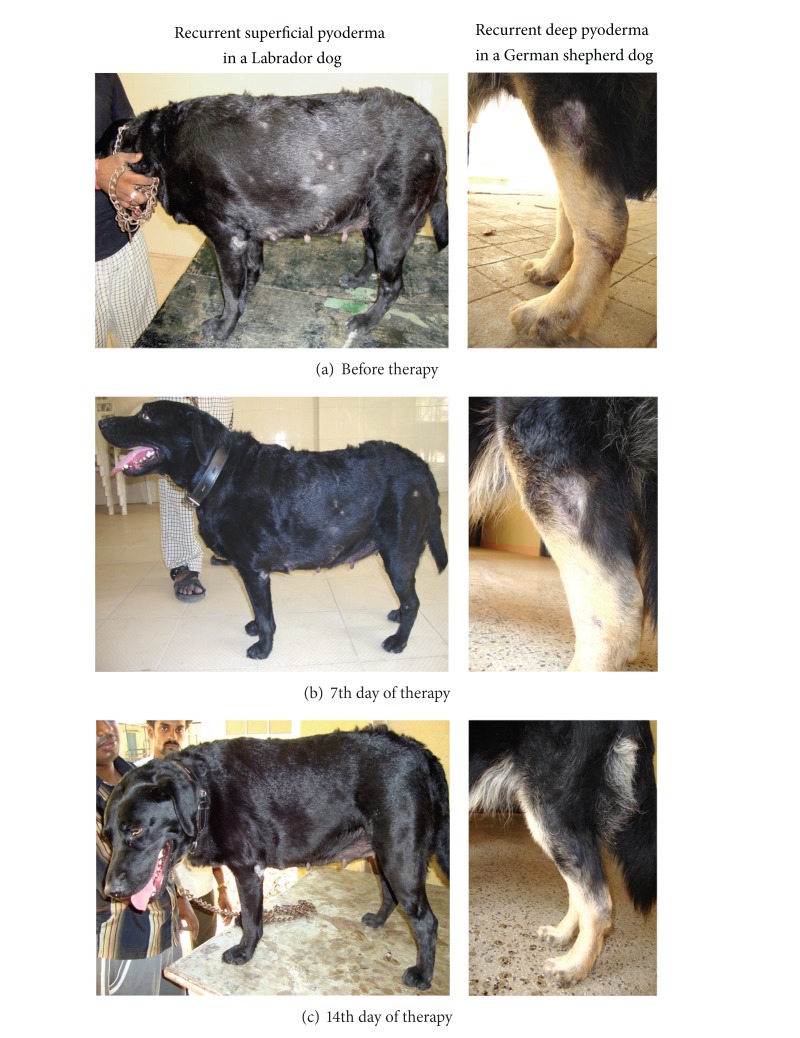
Path of recovery of recurrent pyoderma with cefpodoxime and clavulanic acid.

Out of thirteen dogs with initiated therapy, ten dogs could be monitored fully with complete recovery in all of them indicating that this antibiotic was 100 per cent efficacious. Observations of clinical recovery made at weekly intervals are presented in Table 2. Seven cases (cases 2, 3, 4, 5, 6, 7, and 9) of superficial recurrent pyoderma improved significantly by seventh day, as they were free from the lesions such as papules, crusted papules, and pustules. However, complete recovery was evident by fourteenth day with disappearance of even secondary lesions like erythema, crusts, hyperpigmentation, scales, and so forth. One case (case 1) of superficial pyoderma exhibited only some response to therapy by seventh day with resolution of primary and secondary lesions on 14th and 21st days of therapy (Figure 1). Variation exhibited in the duration (i.e., 2-3 weeks as per clinical observation or as per owner statement days 9–19) of response by the dogs with recurrent superficial pyoderma might be due to variation in the extent of lesions, response of the associated conditions, and the inability to identify the underlying factor and thus its treatment.

Two dogs with deep recurrent pyoderma (cases 8 and 10) showed only some improvement by the end of 7 days with the presence of deep pustules, folliculitis, and ulcers. Though these lesions healed by two weeks, secondary lesions such as erythema, hyperpigmentation, and pruritus were still observed in this dog. However, complete recovery was observed with the continuation of antibiotics for 21 days. In another case of recurrent deep pyoderma (case 10), with generalized demodicosis as an underlying factor, resolution of primary and secondary lesions was observed only on 21 and 28 days of therapy, respectively. Prolonged recovery time in this case could be due to the severity and extent of the lesions and the longer time taken for eliminating the *Demodex*.

These findings are in agreement with Kuhl [11] who stated that most superficial pyodermas require at least three weeks of systemic antibiotics while the duration of antibiotic therapy for deep pyodermas is highly variable and they require long term therapy. All the dogs were monitored for recurrence for a period of six months. Out of ten dogs of this group, in one dog (superficial pyoderma) recurrence was observed 45 days following the end of treatment. Recurrence in this dog could be due to the associated condition of hypothyroidism which was confirmed through laboratory findings after completion of antibiotic therapy. (In this study all the sera samples of dogs were pooled and checked for total T_4_ and free T_4_ levels at a time through ELISA, as the work forms a part of postgraduate study.) However, the recurring condition was treated with the same antibiotic, at the same dosage but along with levothyroxine at 20 *μ*g/kg body weight twice daily. Efficacy of cefpodoxime as reported by previous workers (Cherni et al. [7]) was 96.8%. No adverse effects were seen with this antibiotic in the present study.

In conclusion, this study suggested that cefpodoxime with clavulanic acid is safe and effective in the treatment of recurrent superficial and recurrent deep pyoderma in dogs. The once daily dosing makes cefpodoxime with clavulanic acid a very convenient antibiotic for dog owners, which should increase compliance. Addressing the underlying factors and following proper dose and duration of the antibiotic might have prevented recurrence of the disease in the present study.

## Figures and Tables

**Table 1:** Clinical data of 13 dogs with recurrent pyoderma.

Case no.	Breed	Type of recurrent pyoderma	Sex	Age (Y.M)	Duration of infection (months)	Previous antibiotic therapy	Concurrent dermatosis
1	Labrador	Superficial	F	5.2	12	Lincomycin, enrofloxacin	Hypothyroidism
2	Labrador	Superficial	M	1.2	4	Cephalexin, amoxicillin clavulanate	Demodicosis
3	Lhasa Apso	Superficial	F	5.2	6	Lincomycin	Flea allergic dermatitis
4	Lhasa Apso	Superficial	M	4	6	Enrofloxacin, cephalexin	Hypothyroidism
5	Pug	Superficial	F	4	18	Enrofloxacin, cephalexin	Demodicosis
6	Dachshund	Superficial	F	3.5	4	Amikacin, enrofloxacin	Sarcoptic mange, tick infestation
7	German shepherd	Superficial	F	1		Amoxicillin clavulanate	Flea allergic dermatitis
8	German shepherd	Deep	M	2.5	3	Enrofloxacin	Seborrhea
9	Doberman	Superficial	M	3.2	4	Lincomycin, enrofloxacin	*Malassezia* dermatitis
10	Rottweiler	Deep	M	1	3	Cephalexin, amoxicillin + sulbactam	None identified
11	German shepherd	Superficial	F	8	5	Amoxicillin, cloxacillin	None identified
12	Nondescript	Deep	F	6	8	Amoxicillin clavulanate	*Malassezia* dermatitis, tick infestation
13	Bullmastiff	Superficial	M	1.5	4	Enrofloxacin	*Malassezia* dermatitis

F: female.

M: male.

Y: years.

M: months.

**Table 2:** Therapeutic response to cefpodoxime + clavulanic acid in dogs with recurrent pyoderma.

Case no.	Response exhibited (on day)			Exact time taken for complete recovery as per owners' statement	Relapse
	7	14	21		
1	F	G	E	19	No
2	G	E		12	No
3	G	E		14	Yes (30 days)
4	G	E		9	No
5	G	E		12	No
6	G	E		13	No
7	G	E		10	No
8	F	G	E	17	No
9	G	E		11	No
10	F	G	E	21	No
11	Dropped in the middle of therapy				
12	Dropped in the middle of therapy				
13	Dropped in the middle of therapy				

Clinical response:

E: excellent, complete remission of clinical signs of recurrent pyoderma and point of recovery.

G: good, most primary lesions have resolved but mild secondary lesions such as erythema, crusts, and scales are still evident.

F: fair, some response to treatment but primary and secondary lesions are still evident.
